# Central nervous system hemorrhage related to protein kinase inhibitors: A real-world pharmacovigilance case-control study and genetic correlation study

**DOI:** 10.1097/MD.0000000000051382

**Published:** 2026-11-06

**Authors:** Lin Zhang, Zhexuan Zhao, Yingying Shu, Jianwu Zhang

**Affiliations:** a Shuiguohu Street Community Health Service Center of Wuchang District, Wuhan (Wuchang District Health Education Center of Wuhan), Wuhan, Hubei, China; b Student, School of Public Health, Wuhan University, Wuhan, Hubei, China; c Emergency Center, Zhongnan Hospital of Wuhan University, Wuhan, Hubei, China; d Department of Laboratory Medicine, Zhongnan Hospital of Wuhan University, Wuhan, Hubei, China.

**Keywords:** central nervous system hemorrhage, pharmacovigilance, protein kinase inhibitors

## Abstract

Despite the widespread clinical use of protein kinase inhibitors in oncology, evidence regarding their potential to induce central nervous system (CNS) hemorrhage remains limited. This study aims to investigate the occurrence and risk signals of CNS hemorrhagic events associated with different PKI subclasses, identify the most affected patient populations, and examine variations in risk profiles across demographic subgroups. We conducted disproportionality analyses using data from the FDA Adverse Event Reporting System to detect CNS hemorrhage signals linked to 14 PKI classes. Subgroup analyses stratified by age, sex, and body weight were performed to explore differential risk patterns. Furthermore, Mendelian Randomization and genetic correlation analyses were utilized to evaluate potential causal relationships between PKI therapeutic indications and CNS hemorrhages. We analyzed 9215 CNS hemorrhage reports linked to 14 PKI classes. We identified 6 CNS hemorrhage related signal of disproportionate reporting significantly associated with PKI use. Both BCR-ABL tyrosine kinase inhibitors and BTK inhibitors showed consistent positive signals across all 6 signal of disproportionate reportings, with BTK inhibitors presenting analysis the highest risks (e.g., cerebral hemorrhage reporting odds ratio = 3.30 [3.00–3.62], subdural hematoma reporting odds ratio = 5.90 [5.28–6.60]). Mendelian Randomization revealed no causal genetic relationship between PKI indications and CNS hemorrhage, suggesting the hemorrhagic risks are likely attributable to the drugs themselves rather than underlying diseases. Significant disproportionality signals of PKI-related CNS hemorrhage were identified, while causal links between indications and adverse events were excluded. These findings enhance understanding of PKI-associated bleeding risks and support early detection, targeted monitoring, and informed clinical decision-making.

## 1. Introduction

Since the beginning of the 21st century, cancer treatment has undergone a dramatic transformation, leading to substantial improvements in patient prognosis.^[1]^ Protein kinase inhibitors (PKIs) have achieved remarkable progress in oncology, with their efficacy in significantly prolonging survival widely demonstrated.^[2]^ By selectively inhibiting key intracellular signaling pathways in tumor cells, PKIs suppress tumor growth, proliferation, and metastasis, and have become a cornerstone in the treatment of various malignancies.^[3]^ However, with their increasing clinical use, concerns about the potential safety risks of PKIs have grown, particularly with respect to the central nervous system (CNS). A notable example is the occurrence of CNS hemorrhage during treatment with bevacizumab.^[4]^ Although CNS hemorrhage remains a relatively rare event, it can result in serious neurological deficits and may be life-threatening when it occurs.^[5]^

Existing studies on PKIs have primarily focused on clinical trials. While these studies provide critical evidence for the clinical use of PKIs, they often exclude patients at high risk of cerebral hemorrhage.^[6]^ This selective inclusion may limit the generalizability of clinical trial findings and may not fully reflect the safety profile of PKIs in real-world settings. Real-world pharmacovigilance studies directly address this limitation by utilizing large-scale spontaneous reporting databases to evaluate drug safety across diverse patient populations.^[7]^ For instance, recent disproportionality analyses based on such databases have successfully characterized the risks of neuropsychiatric adverse events, cardiotoxicity, and gastrointestinal bleeding associated with various tyrosine kinase inhibitors(TKIs),^[8–10]^ highlighting the utility of this approach in complementing trial evidence.

In addition to conventional pharmacovigilance methods, Mendelian Randomization (MR): a technique that leverages genetic variants to assess causal relationships: has recently been applied to drug safety research.^[11–13]^

CNS hemorrhage was selected as the critical outcome for this investigation due to its severe clinical consequences, including permanent neurological disability and high mortality, which can dramatically alter the risk-benefit calculus of anticancer therapy.^[4,5]^ However, evidence regarding PKI-associated CNS hemorrhage is limited and fragmented. Existing pharmacovigilance studies have largely focused on other organ toxicities,^[8–10]^ leaving a significant gap in the systematic, class-wide evaluation of CNS bleeding risks. Furthermore, it remains unclear whether any observed risk is attributable to the pharmacological action of PKIs or confounded by the underlying malignancies they treat. To address these gaps, this study was designed to comprehensively evaluate the association between diverse PKI classes and CNS hemorrhage, and to disentangle drug effects from disease-related risk using genetic evidence.

This study aims to address the following key questions: Which types of CNS hemorrhages are more likely to be induced by PKIs? Which subclasses of PKIs are more strongly associated with CNS hemorrhage? Do PKI-associated CNS hemorrhages predominantly or exclusively occur in specific patient subgroups?We prespecified the following hypotheses: there would be heterogeneity in CNS hemorrhage risk across different PKIs, and the risk profile would vary across different demographic subgroups. We conducted a pharmacovigilance study based on adverse event reports (AEs) from the FDA Adverse Event Reporting System (FAERS) to investigate the CNS hemorrhagic risks associated with different PKI subclasses across varying patient populations. Additionally, we employed MR to examine the genome-wide causal relationship between PKI indications and CNS hemorrhage. By integrating large-scale pharmacovigilance data with MR findings, this study provides a comprehensive assessment of the CNS hemorrhagic risks linked to PKIs, offering evidence to guide clinical decision-making and informing future drug safety research. The study design is illustrated in Figure 1.

**Figure 1.**
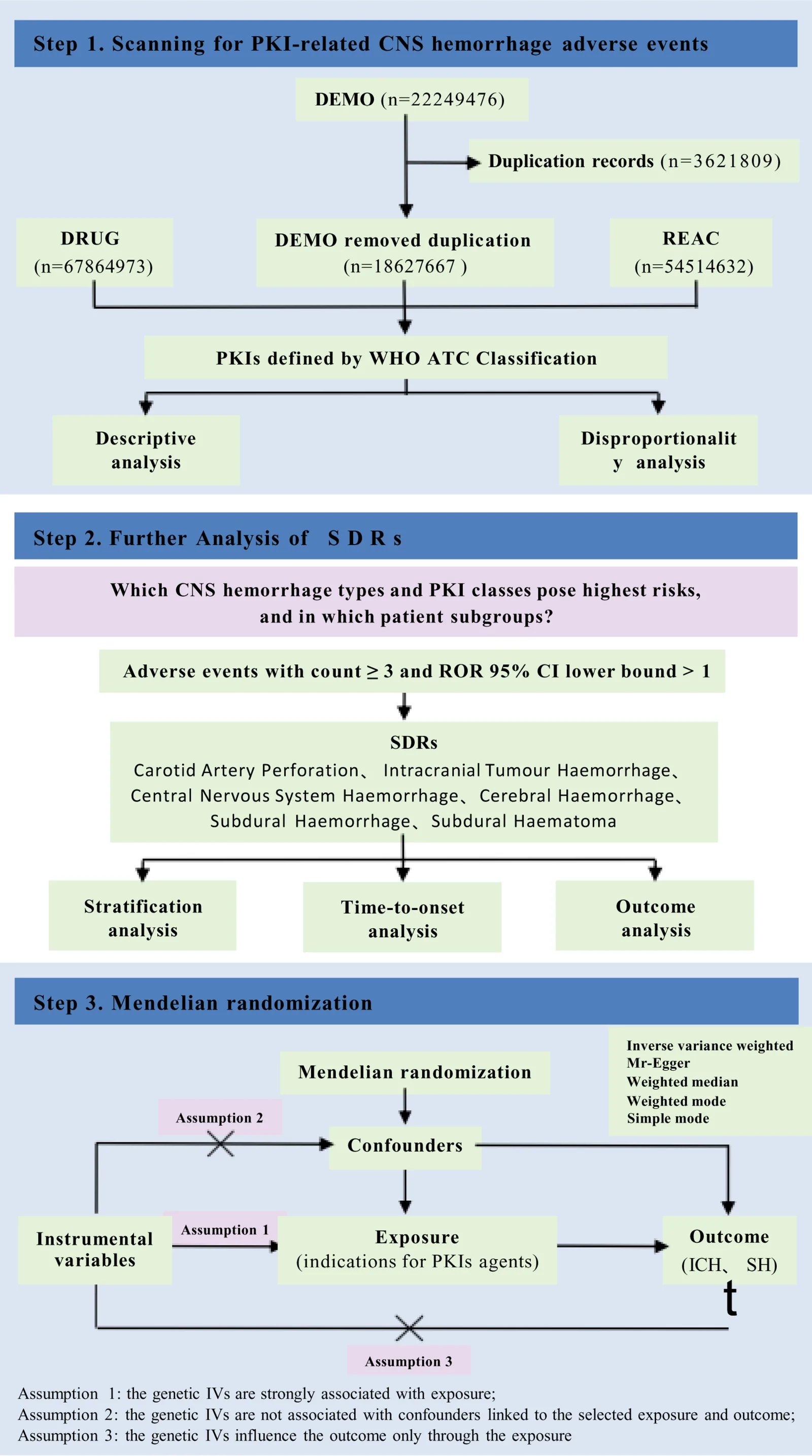
Study design. CNS = central nervous system, CI = confidence interval, DEMO = demographic and administrative information, DRUG = detailed drug-related information, ICH = intracerebral hemorrhage, IVs = instrumental variables, PKI = protein kinase inhibitors, REAC = preferred terms for adverse events, ROR = reporting odds ratio, SDRs = signal of disproportionate reportings, SH = subdural hemorrhage. *n denotes the number of reports, with each patient potentially contributing ≥1 record.

## 2. Methods

### 2.1. Data source and data cleaning

This was a case-control study using the FAERS database, with cases defined as CNS hemorrhage reports and controls as all other AEs.We collected AEs from the FAERS database spanning from January 2004 to December 2024. Data extraction was performed on January 2025. All reports from January 2004 (the inception of the modern FAERS database) through the most recent complete quarter available at that time (Q4 2024) were retrieved and included in the analysis. This timeframe was selected to encompass the maximum contiguous period of data available for analysis at the study’s outset.The classification of antineoplastic agents was based on information from the World Health Organization Collaborating Centre for Drug Statistics Methodology (https://atcddd.fhi.no/) and the National Center for Biotechnology Information (https://www.ncbi.nlm.nih.gov/) classes of PKIs were investigated in this study. These agents represent a broad range of therapeutic mechanisms, enabling a comprehensive evaluation of CNS hemorrhagic risk across diverse treatment categories.

Following FDA guidance, we utilized the deduplicated and standardized version of the FAERS dataset. The data processing followed these steps: First, for entries with the same CASEID, we retained the record with the latest FDA_DT. Second, when CASEID and FDA_DT were identical, the record with the highest PRIMARYID was selected. Only reports where the drug was designated as the primary suspect were included in our analysis. Additionally, we excluded records with no associated AEs. Detailed drug categories and compound names are listed in Supplementary File 1, Supplemental Digital Content 1. Adverse events in the FAERS database were classified using preferred terms (PTs) from the Medical Dictionary for Regulatory Activities version 25.0 (MedDRA). During retrieval, we limited AE reports to PTs under the Standardized The Medical Dictionary for Regulatory Activities version 25.0 Query (The Standardized MedDRA Query) for “Haemorrhagic CNS vascular disorders.”The full list of included PTs is provided in Supplementary File 2, Supplemental Digital Content 2.

The FAERS database utilized in this study consists of publicly available, fully de-identified reports. Prior to public release, the FDA removes all direct personal identifiers from the reports in accordance with its privacy protection policies. In this analysis, we solely accessed and analyzed these preprocessed, aggregated data at the case level. No attempts were made, nor was it possible, to reidentify any individual from the dataset. Therefore, this research did not involve interaction with human subjects or access to private health information, and as such, did not require approval from an institutional review board or ethics committee.All eligible reports during the study period were included.

### 2.2. Statistical analysis of pharmacovigilance analysis

In pharmacovigilance studies, disproportionality can be observed when a specific AE is reported more frequently in association with a particular drug or therapy. In this study, we used the reporting odds ratio (ROR) to detect signals of CNS hemorrhage in FAERS reports. A disproportionality analysis was conducted using the entire FAERS database as the comparator. A signal was considered to indicate a potentially high risk if the number of AEs for a given drug was ≥3 and the lower bound of the 95% confidence interval (CI) of the ROR exceeded 1.^[14]^ PTs meeting these criteria were defined as PKI-related signal of disproportionate reporting (SDR). ROR was also used to compare AEs risk across drugs, with higher RORs indicating greater relative risk.^[15]^The specific ROR calculation method is detailed in Supplementary File 3, Supplemental Digital Content 3. Reports identified as PKI-related SDRs were included for further analysis.To explore subgroup-specific risks of CNS hemorrhage, we stratified the data by age group, sex, and body weight.Age was categorized into 4 groups: 0 to 18, 18 to 65,65 to 85 and 85 to 100 years,based on previous pharmacovigilance studies and biological plausibility. Body weight was grouped as <50 kg, 50 to 100 kg, and >100 kg according to World Health Organization classifications.

The interval between the initiation of a PKI and the reporting of CNS hemorrhage was considered as time to onset (TTO).To assess phenotypic consequences, we evaluated the frequency of serious clinical outcomes associated with CNS hemorrhage. For overlapping outcomes reported under the same patient ID, only the most serious outcome was considered, ranked in descending order of severity as follows: death > life-threatening events > disability > hospitalization (HO) > OT.We estimated the cumulative probability of PKI-related SDRs TTO using the Kaplan–Meier method. The log-rank test and nonparametric Kruskal–Wallis test were used to compare the median TTO between different groups. As all cases experienced the event of interest and fatal outcomes occurred after the onset of PKI-related SDRs, censoring and competing risks were not considered in the analysis.Records with missing age, sex, or weight were excluded from the respective subgroup analyses; no imputation was performed

### 2.3. MR analysis and genetic correlation analysis

This study employed genetic variants associated with exposures as instrumental variables to investigate the potential causal relationships between risk factors and outcomes.^[16]^To fulfill the first assumption, we selected single nucleotide polymorphisms (SNPs) associated with each trait at a genome-wide significance threshold of *P* < 5 × 10^−5^. We retained only SNPs with large physical distances (≥10,000 kb) and low linkage disequilibrium (*R*^2^ < 0.001) to ensure independence among instruments.^[17]^To explore the potential causal relationships between 4 drug indications and the risk of intracerebral hemorrhage (ICH) or subdural hemorrhage (SH), we applied 5 MR methods: inverse variance weighted (IVW), MR Egger, weighted median, simple mode, and weighted mode.^[18–20]^ Among these, the IVW method is considered the most reliable estimator when all selected SNPs are valid instruments.^[21]^Cochran’s *Q* test was used to assess heterogeneity, while MR Egger and IVW regression analyses were employed to evaluate horizontal pleiotropy for genetic instruments involving 2 or more SNPs. A *P*-value < .05 was considered statistically significant. Details of the genome-wide association study data used for MR analysis and genetic correlation analysis are provided in Supplementary Files 4 and 5, Supplemental Digital Content 4.

All data management and statistical analyses for both the FAERS-based pharmacovigilance study and the MR study were conducted using R (version 4.3.0).

## 3. Results

### 3.1. Descriptive analysis for CNS hemorrhage related to PKIs

A total of 9215 reports of CNS hemorrhage associated with the use of 14 PKIs were collected. The 3 most frequently reported PKI classes were: OT, PKIs (n = 2368, 25.70%), BTK inhibitors (n = 1895, 20.56%), BCR-ABL TKIs(n = 1660, 18.01%).When comparing CNS hemorrhage events to the total number of AEs for each drug, the 3 PKI classes with the highest proportion of CNS hemorrhage were: BTK inhibitors (2.51%), BCR-ABL TKIs (1.76%), OT PKIs (1.43%).Overall, CNS hemorrhage-related adverse events occurred most frequently in patients aged 65 to 85 years (n = 3374, 36.60%), weighing 50 to 100 kg (n = 1733, 18.80%), and were more commonly reported in males (n = 4049, 43.90%). The most common reported outcome was prolonged HO (n = 3454, 37.40%) (Fig. 2D).The remaining 7 drug classes were most frequently associated with prolonged HO, especially VEGFR TKIs (n = 90, 45.00%), HER2 TKIs (n = 27, 43.55%), and OT, PKIs (n = 1029, 43.45%) (Fig. 2C).

**Figure 2.**
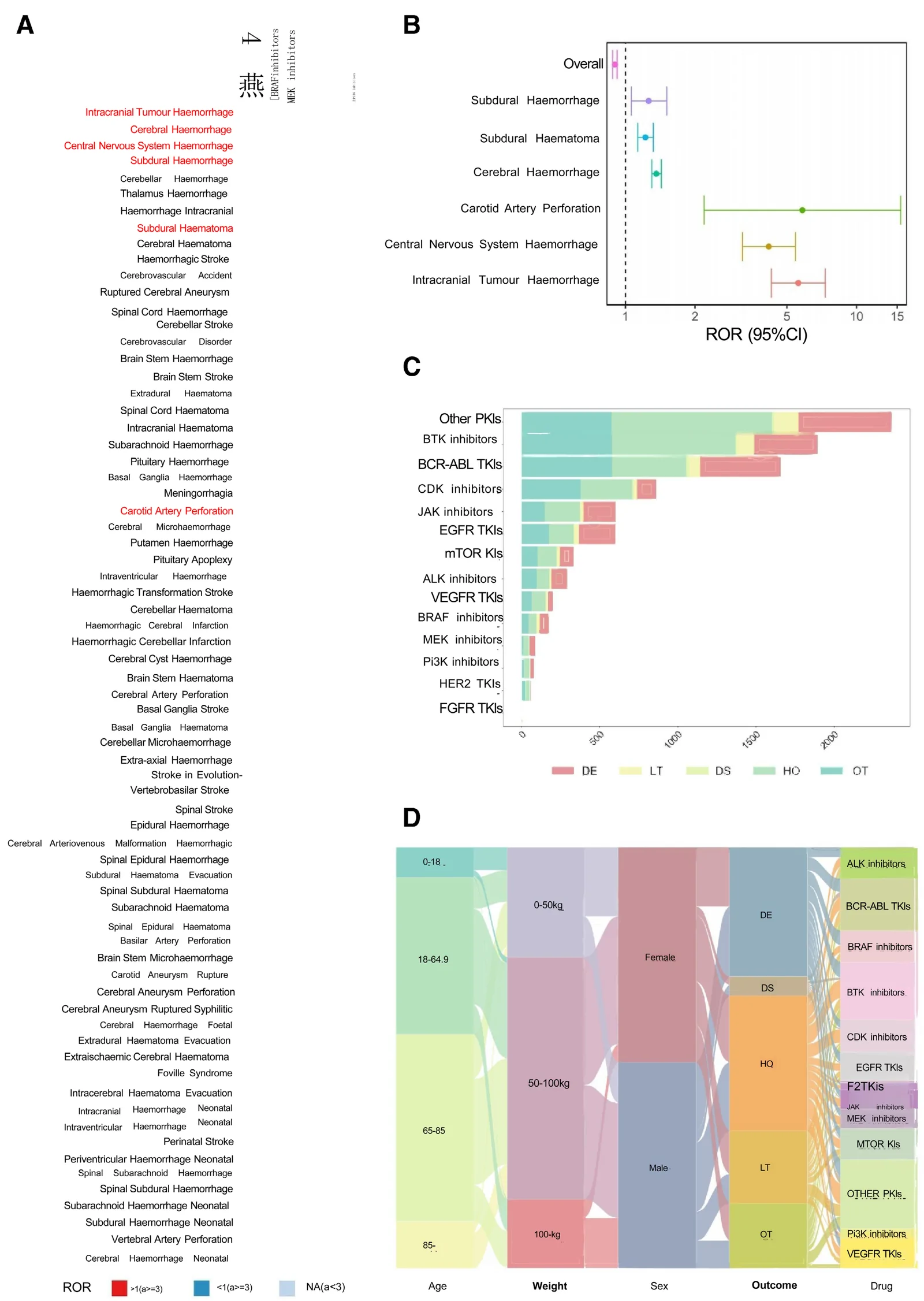
Central nervous system hemorrhage events associated with protein kinase inhibitors based on the FAERS database. (A) Heatmap illustrating the reporting odds ratios (RORs) of CNS hemorrhage-related preferred terms (PTs) under different PKI treatment strategies (only PTs with ≥ 3 cases are shown). Red indicates ROR > 1; dark blue indicates ROR < 1; light blue represents PTs with fewer than 3 cases. PTs highlighted in dark red meet both criteria: the lower bound of the 95% confidence interval (CI) of the ROR exceeds 1, and the number of cases is ≥3. (B) Forest plot showing RORs (with 95% CIs) for 6 significantly associated adverse events and the overall CNS hemorrhage risk. (C) Bar chart displaying the number and proportion of various clinical outcomes under different treatment strategies. (D) Sankey diagram illustrating the distribution and relationship among age groups, body weight, sex, clinical outcomes, and treatment strategies for patients with PKI-related CNS hemorrhage events.

Detailed clinical characteristics of patients experiencing CNS hemorrhage-related to PKI use are provided in Supplementary File 6, Supplemental Digital Content 6.

### 3.2. Scanning for PKI-related CNS hemorrhage adverse events

By calculating the ROR values for individual PTs under different treatment strategies, we identified the PTs most strongly associated with specific PKI therapies. Among events with at least 3 reports, the highest ROR was observed for intracranial tumor hemorrhage following treatment with BRAF inhibitors (n = 16, ROR [95% CI] = 58.19 [35.23–96.14]). This was followed by intracranial tumor hemorrhage associated with MEK inhibitors (n = 7, ROR [95% CI] = 48.62 [23.00–102.78]) and pituitary apoplexy associated with BCR-ABL TKIs (n = 17, ROR [95% CI] = 26.60 [16.02–44.16]) (Fig. 2A).Additionally, across the entire FAERS dataset, we identified 6 PTs that demonstrated a strong disproportionality signal with PKI use: carotid artery perforation (n = 5, ROR [95% CI] = 5.83 [2.19–15.53]), intracranial tumor hemorrhage (n = 66, ROR [95% CI] = 5.60 [4.28–7.32]), CNS hemorrhage (n = 65, ROR [95% CI] = 4.17 [3.21–5.44]), cerebral hemorrhage (n = 1801, ROR [95% CI] = 1.36 [1.30–1.43]), SH (n = 131, ROR [95% CI] = 1.26 [1.06–1.51]), and subdural hematoma (n = 659, ROR [95% CI] = 1.22 [1.13–1.32]) (Fig. 2B).

For the purposes of further analysis, we defined these 6 PTs as SDRs.We subsequently conducted a detailed analysis of the 6 identified SDRs. Among these, the most frequent serious outcome was DE in patients with Carotid Artery Perforation (3 cases, 75.00%), CNS Haemorrhage (26 cases, 59.09%), Cerebral Haemorrhage (364 cases, 40.40%), and Subdural Haemorrhage (26 cases, 39.39%). For Intracranial Tumour Haemorrhage, the most common serious outcome was categorized as OT (12 cases, 41.38%), while for Subdural Haematoma, the most frequent outcome was HO (184 cases, 41.63%) (Fig. 3A).When comparing SDRs to non-SDR adverse events, the most common TTO for SDRs was within 0 to 30 days (335 cases, 36.53%), which was comparable to that of non-SDRs (54,457 cases, 34.71%) (Fig. 3B). The median TTO for fatal outcomes was 58 days, while that for nonfatal outcomes was 84 days; however, this difference was not statistically significant (*P* = .841) (Fig. 3C).Importantly, there were significant differences in median TTO among different SDRs (*P* = .012), with Subdural Haematoma showing the longest median TTO (86 days [IQR 24−292]) and Subdural Haemorrhage the shortest (19 days [IQR 9−136]) (Fig. 3D). Additionally, a significant difference was observed in TTO among different treatment strategies (*P* < .001), with JAK inhibitors associated with the longest median TTO (140 days [IQR 46−270]) and HER2 TKIs the shortest (9 days [IQR 9−9]) (Fig. 3E).

**Figure 3.**
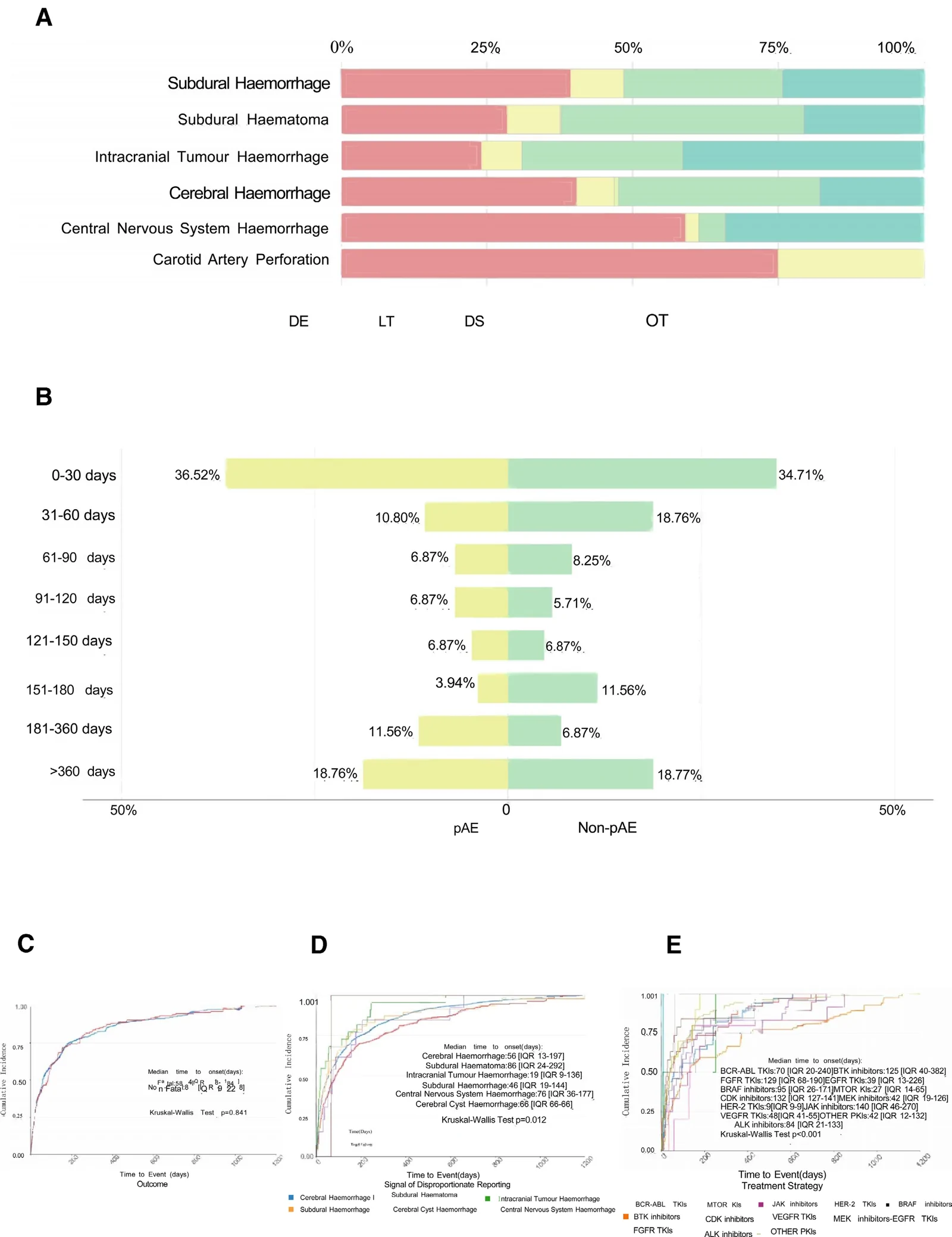
Further analysis of central nervous system hemorrhage events associated with PKI treatment. (A) Bar chart showing the proportion of clinical outcomes for each of the 6 signal of disproportionate reportings (SDRs). (B) Time to onset distribution for SDRs compared with non-PKI-related adverse events. (C–E) From left to right: cumulative distribution curves illustrating the time to onset of PKI-related SDRs stratified by clinical outcome (C), specific SDR type (D), and treatment strategy (E). Statistical significance was assessed using the Kruskal–Wallis test.

### 3.3. Disproportionality analysis for CNS hemorrhage associated with PKIs

Figure 4 presents the signal detection results of CNS hemorrhagic events associated with various treatment strategies. When PTs were restricted to all CNS hemorrhage-related AEs, only BCR-ABL TKIs (n = 1713, ROR [95% CI] = 1.29 [1.23–1.36]) and BTK inhibitors (n = 2017, ROR [95% CI] = 1.87 [1.79–1.95]) showed statistically significant signals.Among specific SDRs, BTK inhibitors exhibited significant signals for cerebral hemorrhage (n = 447, ROR [95% CI] = 3.30 [3.00–3.62]), subdural hematoma (n = 321, ROR [95% CI] = 5.90 [5.28–6.60]), SH (n = 58, ROR [95% CI] = 5.51 [4.25–7.15]), and CNS hemorrhage (n = 29, ROR [95% CI] = 17.20 [11.80–25.08]). BCR-ABL TKIs also showed significant signals for cerebral hemorrhage (n = 225, ROR [95% CI] = 1.35 [1.18–1.54]), subdural hematoma (n = 108, ROR [95% CI] = 1.60 [1.32–1.93]), subdural hemorrhage (n = 26, ROR [95% CI] = 2.00 [1.36–2.94]), and CNS hemorrhage (n = 10, ROR [95% CI] = 4.64 [2.48–8.69]).

**Figure 4.**
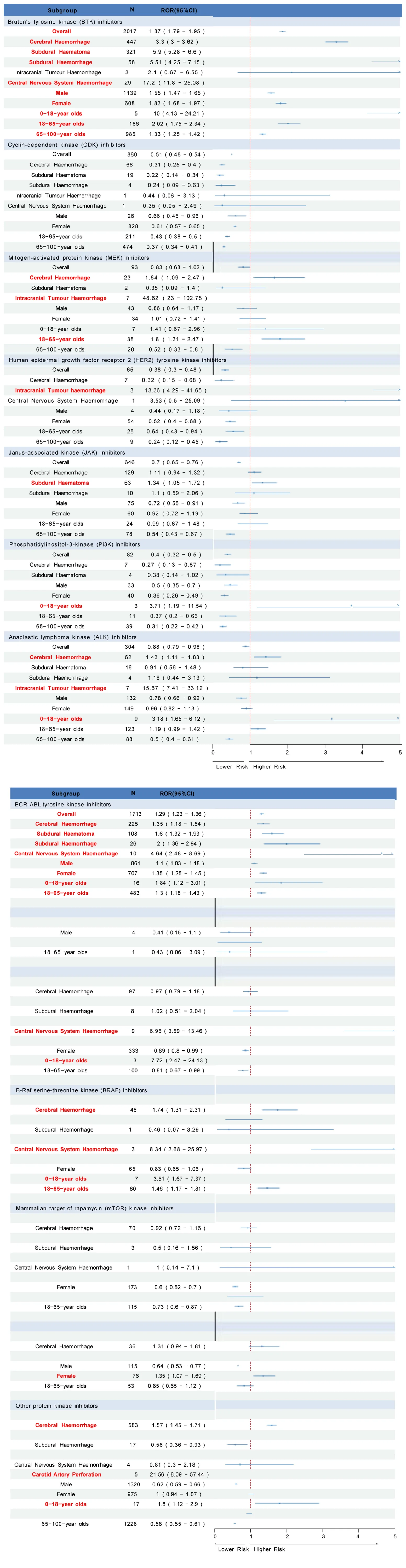
Signal detection results related to central nervous system hemorrhage after different treatment regimens. *N indicates the number of reports; each patient may contribute ≥1 record.

Among all regimens associated with CNS hemorrhage, the highest ROR was observed for BTK inhibitors (n = 29, ROR [95% CI] = 17.20 [11.80–25.08]), followed by BRAF inhibitors (n = 3, ROR [95% CI] = 8.34 [2.68–25.97]) and EGFR TKIs (n = 9, ROR [95% CI] = 6.95 [3.59–13.46]). For cerebral hemorrhage, BTK inhibitors again had the highest ROR (n = 447, ROR [95% CI] = 3.30 [3.00–3.62]), followed by BRAF inhibitors (n = 48, ROR [95% CI] = 1.74 [1.31–2.31]) and MEK inhibitors (n = 23, ROR [95% CI] = 1.64 [1.09–2.47]). For intracranial tumor hemorrhage, the highest signal was seen with BRAF inhibitors (n = 16, ROR [95% CI] = 58.19 [35.23–96.14]), followed by MEK inhibitors (n = 7, ROR [95% CI] = 48.62 [23.00–102.78]) and ALK inhibitors (n = 7, ROR [95% CI] = 15.67 [7.41–33.12]).Significant signals for subdural hemorrhage were observed only with BTK inhibitors (n = 58, ROR [95% CI] = 5.51 [4.25–7.15]) and BCR-ABL TKIs (n = 26, ROR [95% CI] = 2.00 [1.36–2.94]). For subdural hematoma, significant signals were detected with BCR-ABL TKIs (n = 108, ROR [95% CI] = 1.60 [1.32–1.93]), BTK inhibitors (n = 321, ROR [95% CI] = 5.90 [5.28–6.60]), and JAK inhibitors (n = 63, ROR [95% CI] = 1.34 [1.05–1.72]). Carotid artery perforation was only significantly associated with “OT, PKIs” (n = 5, ROR [95% CI] = 21.56 [8.09–57.44]). No significant signal was observed for CDK inhibitors, Pi3K inhibitors, FGFR TKIs, or VEGFR TKIs across any SDR subtypes.

In the male subgroup, the highest ROR was associated with BTK inhibitors (n = 1139, ROR [95% CI] = 1.55 [1.47–1.65]), and similarly, BTK inhibitors also showed the highest ROR in the female subgroup (n = 608, ROR [95% CI] = 1.82 [1.68–1.97]). Specifically, BTK inhibitors exhibited significant signals in both males (n = 1139, ROR [95% CI] = 1.55 [1.47–1.65]) and females (n = 608, ROR [95% CI] = 1.82 [1.68–1.97]). BCR-ABL TKIs also had significant signals in males (n = 861, ROR [95% CI] = 1.10 [1.03–1.18]) and females (n = 707, ROR [95% CI] = 1.35 [1.25–1.45]). VEGFR TKIs showed a significant signal only in female patients (n = 76, ROR [95% CI] = 1.35 [1.07–1.69]). No OT treatment strategies demonstrated significant signals in either gender subgroup.

BTK inhibitors showed significant signals across all age groups: 0 to 18 years (n = 5, ROR [95% CI] = 10.00 [4.13–24.21]), 18 to 65 years (n = 186, ROR [95% CI] = 2.02 [1.75–2.34]), and 65 to 100 years (n = 985, ROR [95% CI] = 1.33 [1.25–1.42]), with the highest ROR values in each respective subgroup. MEK inhibitors showed a significant signal in the 18 to 65 age group (n = 38, ROR [95% CI] = 1.80 [1.31–2.47]). BCR-ABL TKIs showed significant signals in both the 0 to 18 age group (n = 16, ROR [95% CI] = 1.84 [1.12–3.01]) and the 18 to 65 age group (n = 483, ROR [95% CI] = 1.30 [1.18–1.43]). Likewise, BRAF inhibitors demonstrated significant signals in the 0 to 18 age group (n = 7, ROR [95% CI] = 3.51 [1.67–7.37]) and 18 to 65 age group (n = 80, ROR [95% CI] = 1.46 [1.17–1.81]).Several treatment strategies showed significant signals only in the 0 to 18 age group, including Pi3K inhibitors (n = 3, ROR [95% CI] = 3.71 [1.19–11.54]), ALK inhibitors (n = 9, ROR [95% CI] = 3.18 [1.65–6.12]), EGFR TKIs (n = 3, ROR [95% CI] = 7.72 [2.47–24.13]), and OT, PKIs (n = 17, ROR [95% CI] = 1.80 [1.12–2.90]). No OT treatment strategies showed significant signals in any age subgroup.

### 3.4. Results of genetic correlations and MR analysis

Figure 5 illustrates the causal relationships between certain indications of BTK inhibitors and BCR-ABL TKIs and SDRs. MR analysis showed no significant causal associations between any of the investigated indications and ICH or SH. Instrument validity testing indicated that all Cochran’s *Q* tests and MR-Egger pleiotropy tests met the required standards. Detailed results are provided in Supplementary File 7, Supplemental Digital Content 7.

**Figure 5.**
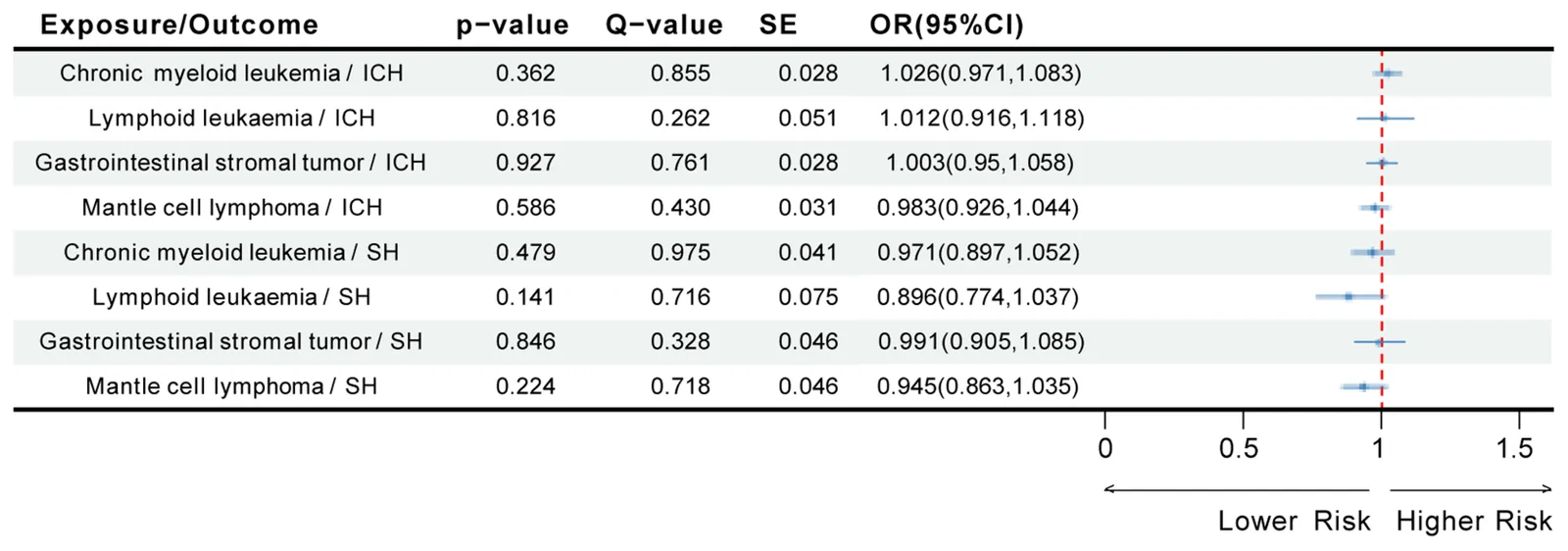
Causal relationships between selected indications of 2 protein kinase inhibitors and SDRs. CI = confidence interval, ICH = intracerebral hemorrhage, SE = standard error, SH = subdural hemorrhage, OR = odds ratio.

## 4. Discussion

Although AEs associated with PKIs have been extensively reported and investigated,^[22–24]^ most existing studies are limited to PKIs with a single mechanism of action, lacking comparative analyses across PKIs with different mechanisms. Moreover, comprehensive investigations into potential CNS hemorrhagic AEs related to PKIs remain scarce.In this study, by integrating real-world pharmacovigilance data with MR evidence, we systematically evaluated the associations between 14 classes of PKIs and CNS hemorrhage. Using the complete FAERS database as a reference, we examined the clinical characteristics of reported cases involving such adverse reactions, identified 6 CNS hemorrhagic events strongly associated with PKI therapy through disproportionality analysis, compared the risks of different SDRs in various populations using stratified analysis, and explored the associations between drug indications and SDRs by incorporating MR evidence.

CNS hemorrhagic events represent a rare but serious category of AEs related to PKIs. In the descriptive analysis, compared with all PTs for each drug, the highest proportion of CNS hemorrhage was observed with BTK inhibitors (1895 cases, 2.51%). These events were also more frequently reported in male patients (4049 cases, 43.90%). A systematic review and meta-analysis summarizing epidemiological trends of ICH over the past 40 years found that men are more likely to develop ICH than women,^[25]^ which is consistent with our findings. Possible explanations include differences in dietary habits, genetic factors, OT variables between sexes.^[26,27]^That review also found that the overall incidence of ICH increases with age, peaking before declining after the age of 85,^[25]^ a pattern also observed in our study. This trend may be attributed to the higher prevalence of hypertension, atherosclerosis, and aneurysms with advancing age, all of which increase the risk of ICH. The subsequent decline in ICH incidence among individuals aged over 85 years may be due to the complex health status of this age group; some patients may die from OT causes, avoid hospital visits for CT scans due to limited mobility, or decline autopsy examinations.^[28]^Additionally, we observed that the most frequently reported weight group was 50 to 100 kg (1733 cases, 18.80%). However, in the absence of disease burden data or studies of OT designs, it cannot be concluded that CNS hemorrhagic events preferentially occur in this group. This finding may also be explained by the base rate fallacy, in which undue attention to the absolute size or observed proportion of a subgroup leads to misinterpretation, without considering its underlying prevalence in the total population.^[29]^ More detailed longitudinal clinical records are needed to further confirm patient characteristics.

Through disproportionality analysis of the FAERS database, we identified 6 PKI-related SDRs. Interestingly, when limiting PTs to all CNS hemorrhage–related AEs, BTK inhibitors (n = 2017, ROR [95% CI] = 1.87 [1.79–1.95]) showed a significant signal. Moreover, BTK inhibitors exhibited the highest risk for CNS hemorrhage (n = 29, ROR [95% CI] = 17.20 [11.80–25.08]), cerebral hemorrhage (n = 447, ROR [95% CI] = 3.30 [3.00–3.62]), subdural hemorrhage (n = 58, ROR [95% CI] = 5.51 [4.25–7.15]), and subdural hematoma (n = 321, ROR [95% CI] = 5.90 [5.28–6.60]).Due to the upstream and central role of BTK in the BCR signaling pathway, combined with its limited expression in OT cell types, BTK represents an attractive therapeutic target, and BTK inhibitors have gradually become the standard treatment for various B-cell malignancies.^[30]^ A study on ibrutinib reported that the incidence of major bleeding was 4.4% for monotherapy and 3.7% for combination therapy. In the overall ibrutinib dataset, 6 fatal major bleeding events were reported, 4 of which were related to CNS hemorrhage (subdural hematoma, intracranial hemorrhage, intraventricular hemorrhage),^[31]^ highlighting the potential for poor outcomes in CNS hemorrhagic events, consistent with our findings.The possible mechanism underlying BTK inhibitor–associated CNS hemorrhage is that BTK, a cytoplasmic nonreceptor agammaglobulinemia tyrosine kinase, is expressed in all hematopoietic lineage cells, particularly B cells, mast cells, and macrophages.^[32]^ Targeted inhibition of BTK can lead to platelet dysfunction,^[33]^ thereby increasing bleeding tendency and ultimately causing CNS hemorrhage. These findings suggest that, for patients receiving BTK inhibitor therapy, CNS hemorrhage risk should be incorporated into the pretreatment assessment. Baseline evaluation should include screening for cerebrovascular disease history, coagulation function, and neuroimaging to identify high risk individuals. Concomitant use of BTK inhibitors with anticoagulant or antiplatelet agents should be strictly avoided; if combination therapy is necessary, enhanced monitoring is recommended.

Similarly, CNS hemorrhagic events associated with BCR-ABL TKIs also warrant attention. In our study, BCR-ABL TKIs showed significant signals for subdural hemorrhage (n = 26, ROR [95% CI] = 2.00 [1.36–2.94]), subdural hematoma (n = 108, ROR [95% CI] = 1.60 [1.32–1.93]), cerebral hemorrhage (n = 225, ROR [95% CI] = 1.35 [1.18–1.54]), and CNS hemorrhage (n = 10, ROR [95% CI] = 4.64 [2.48–8.69]).BCR-ABL is a tyrosine kinase that drives the pathogenesis of chronic myeloid leukemia. Given that tyrosine kinase activity is essential for the transforming ability of BCR-ABL, BCR-ABL TKIs are effective therapies for CML.^[34]^ The FDA has issued warnings regarding nilotinib- and ponatinib-induced bleeding. An in vitro study demonstrated that ponatinib could induce apoptosis and necrosis in human umbilical vein endothelial cells, while dasatinib could inhibit endothelial cell proliferation and impair wound closure; in contrast, imatinib, nilotinib, and bosutinib exerted only minimal effects on endothelial cells.^[35]^ These findings suggest that BCR-ABL TKIs may cause CNS hemorrhage by damaging endothelial cells.Additionally, previous studies have shown that certain BCR-ABL TKIs can directly impair platelet function, which may also contribute to CNS hemorrhage in this setting. Despite these risks, the survival benefits of BCR-ABL TKIs in CML remain irreplaceable. Therefore, in patients with underlying cerebrovascular disease, drugs with lower endothelial toxicity should be considered preferentially, and a tiered prevention strategy should be implemented to reduce the incidence of serious adverse events.

In the MR analysis, we found no significant causal relationship between PKI indications and ICH or subdural hemorrhage (SH). This negative result suggests that the inherent biological pathways of these indications are not independent causal factors for CNS hemorrhage. From a genetic perspective, this finding rules out the underlying disease states themselves as fundamental risk drivers for ICH/SH in patients receiving PKIs, thereby alleviating clinical concerns that “the underlying condition itself substantially triggers treatment-related hemorrhage.”Furthermore, when considered alongside the potential bleeding signals observed in real-world pharmacovigilance studies, our results imply that any increased bleeding risk in clinical practice is more likely attributable to the direct pharmacological effects of the drug,^[36]^ treatment-related complications,^[37]^ or patient-specific factors,^[38]^ rather than solely to the intrinsic pathological mechanisms of the indication. This offers a new perspective for clinical risk stratification: patients with an indication but without traditional bleeding risk factors may not require bleeding prevention measures based solely on the disease itself; instead, attention should focus on monitoring for drug toxicities and managing comorbidities.It should be noted that, due to the absence of The genome-wide association study data for PKI drug targets, we were compelled to use “indications” as a proxy for “drug exposure.” This surrogate measure reflects only the genetic susceptibility to the disease, without directly capturing the causal effect of the drug’s pharmacological action on bleeding risk. This introduces 3 potential sources of bias: indication-related genetic variants may be associated with complications of the disease, acting as confounders; inability to distinguish risk heterogeneity among different drugs for the same indication; and masking of dynamic effects related to treatment intensity or duration. Future studies using MR based on genetic variants of drug targets are warranted to directly elucidate the mechanisms underlying PKI-associated bleeding risk, enabling multidimensional validation in conjunction with real-world pharmacovigilance data.

This study has several limitations. First, the FAERS database is a spontaneous reporting system that may be subject to reporting bias, underreporting, and duplicate entries.^[39]^ Underreporting is likely to bias the ROR estimates toward the null, whereas selective reporting of serious or unexpected CNS hemorrhage events may inflate ROR values. The net direction and magnitude of these biases cannot be precisely quantified with the available data.As some reports have not been verified by healthcare professionals, the reported events cannot be fully attributed to the study drugs, which may affect the generalizability of our findings. Second, the database contains incomplete information regarding dosage, comorbidities, and OT clinical details; therefore, we were unable to evaluate the correlation between biologic dosage and PKI-induced CNS hemorrhage. At present, no widely accepted methods exist to mitigate the influence of these factors on disproportionality analyses.^[40]^In addition, disproportionality analysis in pharmacovigilance studies has limited ability to establish causality.^[41]^ To validate our observations, we conducted MR analysis, which showed that certain indications for BCR-ABL TKIs and BTK inhibitors were not significantly causally related to ICH or subdural hemorrhage. However, the potential association between drug-specific genetic targets and CNS hemorrhage requires further investigation.Future studies should aim to establish large-scale, long-term prospective external cohorts to confirm our results, and should further explore PKI-induced CNS hemorrhage, particularly the underlying pathophysiological mechanisms and patient-specific genetic risk factors, which are crucial for guiding therapeutic decision-making.

## 5. Conclusion

This study elucidates the association between PKI therapy and various adverse events related to CNS hemorrhage, identifying significant disproportionality signals in real-world pharmacovigilance data and ruling out causal relationships between certain indications and SDRs. These findings enhance our understanding of PKI-associated hemorrhagic risk and may aid clinicians in early identification, targeted monitoring, and informed therapeutic decision-making.

## Author contributions

**Data curation:** Lin Zhang, Zhexuan Zhao.

**Formal analysis:** Lin Zhang, Zhexuan Zhao.

**Investigation:** Lin Zhang, Zhexuan Zhao.

**Methodology:** Lin Zhang, Yingying Shu.

**Software:** Lin Zhang, Zhexuan Zhao.

**Validation:** Lin Zhang, Yingying Shu.

**Visualization:** Lin Zhang, Zhexuan Zhao.

**Conceptualization:** Yingying Shu, Jianwu Zhang.

**Funding acquisition:** Yingying Shu, Jianwu Zhang.

**Supervision:** Yingying Shu, Jianwu Zhang.

**Project administration:** Jianwu Zhang.

**Resources:** Jianwu Zhang.

**Writing – original draft:** Lin Zhang, Zhexuan Zhao.

**Writing – review & editing:** Lin Zhang, Zhexuan Zhao, Yingying Shu, Jianwu Zhang.














