# Supplementary material for: Central nervous system hemorrhage related to protein kinase inhibitors: A real-world pharmacovigilance case-control study and genetic correlation study
Source: Medicine (Baltimore). 2026 Nov 6;105(45):e51382. doi: 10.1097/MD.0000000000051382 (PMC13747402; doi:10.1097/MD.0000000000051382)
Supplement: Supplementary file 1 [file medi-105-e51382-s001.docx]

**Supplementary file1. All of product names referred to specific protein kinase inhibitors in FAERS database.**

| **Drug category** | **Drug name** | **ATC code** |
| --- | --- | --- |
| BCR-ABL tyrosine kinase inhibitors | imatinib | L01EA01 |
|  | dasatinib | L01EA02 |
|  | nilotinib | L01EA03 |
|  | bosutinib | L01EA04 |
|  | ponatinib | L01EA05 |
|  | asciminib | L01EA06 |
| Bruton's tyrosine kinase (BTK) inhibitors | ibrutinib | L01EL01 |
|  | acalabrutinib | L01EL02 |
|  | zanubrutinib | L01EL03 |
|  | pirtobrutinib | L01EL05 |
|  | tirabrutinib | L01EL06 |
| Fibroblast growth factor receptor (FGFR) tyrosine kinase inhibitors | erdafitinib | L01EN01 |
|  | pemigatinib | L01EN02 |
|  | infigratinib | L01EN03 |
|  | futibatinib | L01EN04 |
| Epidermal growth factor receptor (EGFR) tyrosine kinase inhibitors | gefitinib | L01EB01 |
|  | erlotinib | L01EB02 |
|  | afatinib | L01EB03 |
|  | osimertinib | L01EB04 |
|  | dacomitinib | L01EB07 |
|  | lazertinib | L01EB09 |
|  | mobocertinib | L01EB10 |
| B-Raf serine-threonine kinase (BRAF) inhibitors | vemurafenib | L01EC01 |
|  | dabrafenib | L01EC02 |
|  | encorafenib | L01EC03 |
| Mammalian target of rapamycin (mTOR) kinase inhibitors | temsirolimus | L01EG01 |
|  | everolimus | L01EG02 |
|  | ridaforolimus | L01EG03 |
|  | sirolimus | L01EG04 |
| Cyclin-dependent kinase (CDK) inhibitors | palbociclib | L01EF01 |
|  | ribociclib | L01EF02 |
|  | abemaciclib | L01EF03 |
| Anaplastic lymphoma kinase (ALK) inhibitors | crizotinib | L01ED01 |
|  | ceritinib | L01ED02 |
|  | alectinib | L01ED03 |
|  | brigatinib | L01ED04 |
|  | lorlatinib | L01ED05 |
| Mitogen-activated protein kinase (MEK) inhibitors | trametinib | L01EE01 |
|  | cobimetinib | L01EE02 |
|  | binimetinib | L01EE03 |
|  | selumetinib | L01EE04 |
| Human epidermal growth factor receptor 2 (HER2) tyrosine kinase inhibitors | lapatinib | L01EH01 |
|  | neratinib | L01EH02 |
|  | tucatinib | L01EH03 |
| Janus-associated kinase (JAK) inhibitors | ruxolitinib | L01EJ01 |
|  | fedratinib | L01EJ02 |
|  | pacritinib | L01EJ03 |
|  | momelotinib | L01EJ04 |
| Vascular endothelial growth factor receptor (VEGFR) tyrosine kinase inhibitors | axitinib | L01EK01 |
|  | cediranib | L01EK02 |
|  | tivozanib | L01EK03 |
|  | fruquintinib | L01EK04 |
| Phosphatidylinositol-3-kinase (Pi3K) inhibitors | idelalisib | L01EM01 |
|  | copanlisib | L01EM02 |
|  | alpelisib | L01EM03 |
|  | duvelisib | L01EM04 |
| Other protein kinase inhibitors | sunitinib | L01EX01 |
|  | sorafenib | L01EX02 |
|  | pazopanib | L01EX03 |
|  | vandetanib | L01EX04 |
|  | regorafenib | L01EX05 |
|  | masitinib | L01EX06 |
|  | cabozantinib | L01EX07 |
|  | lenvatinib | L01EX08 |
|  | nintedanib | L01EX09 |
|  | midostaurin | L01EX10 |
|  | quizartinib | L01EX11 |
|  | larotrectinib | L01EX12 |
|  | gilteritinib | L01EX13 |
|  | entrectinib | L01EX14 |
|  | pexidartinib | L01EX15 |
|  | capmatinib | L01EX17 |
|  | avapritinib | L01EX18 |
|  | ripretinib | L01EX19 |
|  | tepotinib | L01EX21 |
|  | selpercatinib | L01EX22 |
|  | pralsetinib | L01EX23 |
|  | umbralisib | L01EX25 |
|  | capivasertib | L01EX27 |
|  | repotrectinib | L01EX28 |
