# Supplementary material for: Central nervous system hemorrhage related to protein kinase inhibitors: A real-world pharmacovigilance case-control study and genetic correlation study
Source: Medicine (Baltimore). 2026 Nov 6;105(45):e51382. doi: 10.1097/MD.0000000000051382 (PMC13747402; doi:10.1097/MD.0000000000051382)
Supplement: Supplementary file 2 [file medi-105-e51382-s002.docx]

**Supplementary file2.All of the PTs used to describe the central nervous system hemorrhage.**

**PTs**

Basal ganglia haematoma

Basal ganglia haemorrhage

Basal ganglia stroke

Basilar artery perforation

Brain stem haematoma

Brain stem haemorrhage

Brain stem microhaemorrhage

Brain stem stroke

Carotid aneurysm rupture

Carotid artery perforation

Central nervous system haemorrhage

Cerebellar haematoma

Cerebellar haemorrhage

Cerebellar microhaemorrhage

Cerebellar stroke

Cerebral aneurysm perforation

Cerebral aneurysm ruptured syphilitic

Cerebral arteriovenous malformation haemorrhagic

Cerebral artery perforation

Cerebral cyst haemorrhage

Cerebral haematoma

Cerebral haemorrhage

Cerebral haemorrhage foetal

Cerebral haemorrhage neonatal

Cerebral microhaemorrhage

Cerebrovascular accident

Cerebrovascular disorder

Epidural haemorrhage

Extra-axial haemorrhage

Extradural haematoma

Extradural haematoma evacuation

Extraischaemic cerebral haematoma

Foville syndrome

Haemorrhage intracranial

Haemorrhagic cerebellar infarction

Haemorrhagic cerebral infarction

Haemorrhagic stroke

Haemorrhagic transformation stroke

Intracerebral haematoma evacuation

Intracranial haematoma

Intracranial haemorrhage neonatal

Intracranial tumour haemorrhage

Intraventricular haemorrhage

Intraventricular haemorrhage neonatal
