# Supplementary material for: Central nervous system hemorrhage related to protein kinase inhibitors: A real-world pharmacovigilance case-control study and genetic correlation study
Source: Medicine (Baltimore). 2026 Nov 6;105(45):e51382. doi: 10.1097/MD.0000000000051382 (PMC13747402; doi:10.1097/MD.0000000000051382)
Supplement: Supplementary file 3 [file medi-105-e51382-s003.docx]

**Supplementary file3. The methodology for determining ROR in the pharmacovigilance study.**

|  | **Adverse events of targeted disorders** | **All other adverse events** |
| --- | --- | --- |
| **Specific protein kinase inhibitor** | a | b |
| **All other drugs** | c | d |
| **Drugs in total** | a+c | b+d |

ROR=a*d/(b*c)	$95 CI\%=e^{ln(ROR)\pm1.96\sqrt{(1/a+1/b+1/c+1/d)}}$
