# Supplementary material for: Central nervous system hemorrhage related to protein kinase inhibitors: A real-world pharmacovigilance case-control study and genetic correlation study
Source: Medicine (Baltimore). 2026 Nov 6;105(45):e51382. doi: 10.1097/MD.0000000000051382 (PMC13747402; doi:10.1097/MD.0000000000051382)
Supplement: Supplementary file 4 [file medi-105-e51382-s004.docx]

**Supplementary file4. Detailed information of GWAS data**

| **Traits** | **Cases** | **Controls** | **Number of SNPs** | **Source** | **Dataset.id** | **PMID** |
| --- | --- | --- | --- | --- | --- | --- |
| Chronic myeloid leukemia | 111 | 217,902.00 | 16,380,466.00 | FinnGen R10 | finngen_R10_CML | NA |
| Lymphoid leukaemia | 663 | 218,129.00 | 16,380,466.00 | FinnGen R10 | finngen_R10_LYMPHOID_LEUKAEMIA | NA |
| Gastrointestinal stromal tumor | 259 | 314,146.00 | 234,675.00 | FinnGen R10 | finngen_R10_C3_GASTRINT_STROM_EXALLC | NA |
| Mantle cell lymphoma | 210 | 314,193.00 | 206,345.00 | FinnGen R10 | finngen_R10_C3_MANTLE_CELL_LYMPHOMA_EXALLC | NA |
| Intracerebral hemorrhage | 698 | 387,165.00 | 510,645.00 | GWAS Catalog | GCST90080030 | 34662886 |
| Subdural hemorrhage | 244 | 456,104.00 | 11,842,647.00 | GWAS Catalog | GCST90043995 | 34737426 |

*Finngen R10:https://www.finngen.fi/en/access_results; GWAS Catalog:https://www.ebi.ac.uk/gwas/studies
