# Supplementary material for: Central nervous system hemorrhage related to protein kinase inhibitors: A real-world pharmacovigilance case-control study and genetic correlation study
Source: Medicine (Baltimore). 2026 Nov 6;105(45):e51382. doi: 10.1097/MD.0000000000051382 (PMC13747402; doi:10.1097/MD.0000000000051382)
Supplement: Supplementary file 5 [file medi-105-e51382-s005.docx]

**Supplementary file5. The FDA's prescribing information for each biological product**

| **Generic name** | **Brand name** | **Types of biological agents** | **Indications** |
| --- | --- | --- | --- |
| Imatinib | Gleevec | BCR-ABL tyrosine kinase inhibitors | Chronic Myeloid Leukemia,Gastrointestinal Stromal Tumors |
| Dasatinib | Sprycel | BCR-ABL tyrosine kinase inhibitors | Chronic Myeloid Leukemia,Philadelphia Chromosome-Positive Acute Lymphoblastic Leukemia |
| Nilotinib | Danziten | BCR-ABL tyrosine kinase inhibitors | Chronic Myeloid Leukemia,Philadelphia Chromosome-Positive Acute Lymphoblastic Leukemia |
| Bosutinib | Bosulif | BCR-ABL tyrosine kinase inhibitors | Chronic Myeloid Leukemia,Philadelphia Chromosome-Positive Acute Lymphoblastic Leukemia |
| Ponatinib | Iclusig | BCR-ABL tyrosine kinase inhibitors | Chronic Myeloid Leukemia,Philadelphia Chromosome-Positive Acute Lymphoblastic Leukemia |
| Asciminib | Scemblix | BCR-ABL tyrosine kinase inhibitors | Chronic Myeloid Leukemia,Philadelphia Chromosome-Positive Acute Lymphoblastic Leukemia |
| Ibrutinib | Imbruvica | Bruton's tyrosine kinase (BTK) inhibitors | Mantle Cell Lymphoma,Chronic Lymphocytic Leukemia |
| Acalabrutinib | Calquence | Bruton's tyrosine kinase (BTK) inhibitors | Mantle Cell Lymphoma |
| Zanubrutinib | Brukinsa | Bruton's tyrosine kinase (BTK) inhibitors | Mantle Cell Lymphoma |
| Pirtobrutinib | Jaypirca | Bruton's tyrosine kinase (BTK) inhibitors | Mantle Cell Lymphoma |

*Source: https://www.accessdata.fda.gov/scripts/cder/daf/index.cfm
