# Supplementary material for: Central nervous system hemorrhage related to protein kinase inhibitors: A real-world pharmacovigilance case-control study and genetic correlation study
Source: Medicine (Baltimore). 2026 Nov 6;105(45):e51382. doi: 10.1097/MD.0000000000051382 (PMC13747402; doi:10.1097/MD.0000000000051382)
Supplement: Supplementary file 6 [file medi-105-e51382-s006.docx]

**Supplementary file6. Descriptive analysis of central nervous system hemorrhage in specific protein kinase inhibitors users (N denotes the number of participant)**

| **Characteristic** |  |  | **Age（year）** | **＜18** | **18-64.9** | **65-85** | **＞85** | **Missing** | **Weight** | **＜50kg** | **50-100kg** | **＞100kg** | **Missing** | **Sex** | **Female** | **male** | **Missing** |
| --- | --- | --- | --- | --- | --- | --- | --- | --- | --- | --- | --- | --- | --- | --- | --- | --- | --- |
| **BCR-ABL tyrosine kinase inhibitors** | | | | | | | | | | | | | | | | | |
|  | NO_target_PT | (N=92799) |  | 1497 (1.6%) | 30398 (32.8%) | 17770 (19.1%) | 1259 (1.4%) | 41875 (45.1%) |  | 1105 (1.2%) | 8803 (9.5%) | 1128 (1.2%) | 81763 (88.1%) |  | 41097 (44.3%) | 44105 (47.5%) | 7597 (8.2%) |
|  | target_PT | (N=1660) |  | 16 (1.0%) | 418 (25.2%) | 405 (24.4%) | 38 (2.3%) | 783 (47.2%) |  | 20 (1.2%) | 212 (12.8%) | 26 (1.6%) | 1402 (84.5%) |  | 688 (41.4%) | 833 (50.2%) | 139 (8.4%) |
|  | Overall | (N=94459) |  | 1513 (1.6%) | 30816 (32.6%) | 18175 (19.2%) | 1297 (1.4%) | 42658 (45.2%) |  | 1125 (1.2%) | 9015 (9.5%) | 1154 (1.2%) | 83165 (88.0%) |  | 41785 (44.2%) | 44938 (47.6%) | 7736 (8.2%) |
| **Bruton's tyrosine kinase (BTK) inhibitors** | | | | | | | | | | | | | | | | | |
|  | NO_target_PT | (N=73460) |  | 86 (0.1%) | 6957 (9.5%) | 20619 (28.1%) | 2934 (4.0%) | 42864 (58.4%) |  | 732 (1.0%) | 8371 (11.4%) | 1426 (1.9%) | 62931 (85.7%) |  | 25632 (34.9%) | 40362 (54.9%) | 7466 (10.2%) |
|  | target_PT | (N=1895) |  | 4 (0.2%) | 180 (9.5%) | 804 (42.4%) | 119 (6.3%) | 788 (41.6%) |  | 25 (1.3%) | 338 (17.8%) | 40 (2.1%) | 1492 (78.7%) |  | 572 (30.2%) | 1083 (57.2%) | 240 (12.7%) |
|  | Overall | (N=75355) |  | 90 (0.1%) | 7137 (9.5%) | 21423 (28.4%) | 3053 (4.1%) | 43652 (57.9%) |  | 757 (1.0%) | 8709 (11.6%) | 1466 (1.9%) | 64423 (85.5%) |  | 26204 (34.8%) | 41445 (55.0%) | 7706 (10.2%) |
| **Fibroblast growth factor receptor (FGFR) tyrosine kinase inhibitors** | | | | | | | | | | | | | | | | | |
|  | NO_target_PT | (N=1740) |  | 22 (1.3%) | 207 (11.9%) | 297 (17.1%) | 22 (1.3%) | 1192 (68.5%) |  | 25 (1.4%) | 239 (13.7%) | 22 (1.3%) | 1454 (83.6%) |  | 494 (28.4%) | 653 (37.5%) | 593 (34.1%) |
|  | target_PT | (N=7) |  | 0 (0%) | 1 (14.3%) | 2 (28.6%) | 0 (0%) | 4 (57.1%) |  | 0 (0%) | 2 (28.6%) | 1 (14.3%) | 4 (57.1%) |  | 2 (28.6%) | 4 (57.1%) | 1 (14.3%) |
|  | Overall | (N=1747) |  | 22 (1.3%) | 208 (11.9%) | 299 (17.1%) | 22 (1.3%) | 1196 (68.5%) |  | 25 (1.4%) | 241 (13.8%) | 23 (1.3%) | 1458 (83.5%) |  | 496 (28.4%) | 657 (37.6%) | 594 (34.0%) |
| **Epidermal growth factor receptor (EGFR) tyrosine kinase inhibitors** | | | | | | | | | | | | | | | | | |
|  | NO_target_PT | (N=59653) |  | 87 (0.1%) | 10444 (17.5%) | 14809 (24.8%) | 1274 (2.1%) | 33039 (55.4%) |  | 1968 (3.3%) | 6712 (11.3%) | 301 (0.5%) | 50672 (84.9%) |  | 31360 (52.6%) | 20479 (34.3%) | 7814 (13.1%) |
|  | target_PT | (N=598) |  | 2 (0.3%) | 93 (15.6%) | 170 (28.4%) | 14 (2.3%) | 319 (53.3%) |  | 22 (3.7%) | 83 (13.9%) | 0 (0%) | 493 (82.4%) |  | 316 (52.8%) | 221 (37.0%) | 61 (10.2%) |
|  | Overall | (N=60251) |  | 89 (0.1%) | 10537 (17.5%) | 14979 (24.9%) | 1288 (2.1%) | 33358 (55.4%) |  | 1990 (3.3%) | 6795 (11.3%) | 301 (0.5%) | 51165 (84.9%) |  | 31676 (52.6%) | 20700 (34.4%) | 7875 (13.1%) |
| **B-Raf serine-threonine kinase (BRAF) inhibitors** | | | | | | | | | | | | | | | | | |
|  | NO_target_PT | (N=14827) |  | 324 (2.2%) | 5208 (35.1%) | 4087 (27.6%) | 185 (1.2%) | 5023 (33.9%) |  | 356 (2.4%) | 3846 (25.9%) | 460 (3.1%) | 10165 (68.6%) |  | 6196 (41.8%) | 7124 (48.0%) | 1507 (10.2%) |
|  | target_PT | (N=174) |  | 7 (4.0%) | 77 (44.3%) | 49 (28.2%) | 1 (0.6%) | 40 (23.0%) |  | 8 (4.6%) | 64 (36.8%) | 5 (2.9%) | 97 (55.7%) |  | 63 (36.2%) | 95 (54.6%) | 16 (9.2%) |
|  | Overall | (N=15001) |  | 331 (2.2%) | 5285 (35.2%) | 4136 (27.6%) | 186 (1.2%) | 5063 (33.8%) |  | 364 (2.4%) | 3910 (26.1%) | 465 (3.1%) | 10262 (68.4%) |  | 6259 (41.7%) | 7219 (48.1%) | 1523 (10.2%) |
| **Mammalian target of rapamycin (mTOR) kinase inhibitors** | | | | | | | | | | | | | | | | | |
|  | NO_target_PT | (N=37161) |  | 2102 (5.7%) | 11542 (31.1%) | 7270 (19.6%) | 217 (0.6%) | 16030 (43.1%) |  | 1108 (3.0%) | 5987 (16.1%) | 525 (1.4%) | 29541 (79.5%) |  | 19924 (53.6%) | 14111 (38.0%) | 3126 (8.4%) |
|  | target_PT | (N=333) |  | 9 (2.7%) | 109 (32.7%) | 96 (28.8%) | 1 (0.3%) | 118 (35.4%) |  | 10 (3.0%) | 80 (24.0%) | 8 (2.4%) | 235 (70.6%) |  | 166 (49.8%) | 138 (41.4%) | 29 (8.7%) |
|  | Overall | (N=37494) |  | 2111 (5.6%) | 11651 (31.1%) | 7366 (19.6%) | 218 (0.6%) | 16148 (43.1%) |  | 1118 (3.0%) | 6067 (16.2%) | 533 (1.4%) | 29776 (79.4%) |  | 20090 (53.6%) | 14249 (38.0%) | 3155 (8.4%) |
| **Cyclin-dependent kinase (CDK) inhibitors** | | | | | | | | | | | | | | | | | |
|  | NO_target_PT | (N=105463) |  | 108 (0.1%) | 37102 (35.2%) | 40533 (38.4%) | 2926 (2.8%) | 24794 (23.5%) |  | 1469 (1.4%) | 17169 (16.3%) | 1719 (1.6%) | 85106 (80.7%) |  | 97597 (92.5%) | 2229 (2.1%) | 5637 (5.3%) |
|  | target_PT | (N=860) |  | 0 (0%) | 203 (23.6%) | 421 (49.0%) | 45 (5.2%) | 191 (22.2%) |  | 14 (1.6%) | 174 (20.2%) | 12 (1.4%) | 660 (76.7%) |  | 809 (94.1%) | 25 (2.9%) | 26 (3.0%) |
|  | Overall | (N=106323) |  | 108 (0.1%) | 37305 (35.1%) | 40954 (38.5%) | 2971 (2.8%) | 24985 (23.5%) |  | 1483 (1.4%) | 17343 (16.3%) | 1731 (1.6%) | 85766 (80.7%) |  | 98406 (92.6%) | 2254 (2.1%) | 5663 (5.3%) |
| **Anaplastic lymphoma kinase (ALK) inhibitors** | | | | | | | | | | | | | | | | | |
|  | NO_target_PT | (N=25982) |  | 606 (2.3%) | 9712 (37.4%) | 6676 (25.7%) | 332 (1.3%) | 8656 (33.3%) |  | 871 (3.4%) | 5000 (19.2%) | 398 (1.5%) | 19713 (75.9%) |  | 12959 (49.9%) | 10052 (38.7%) | 2971 (11.4%) |
|  | target_PT | (N=291) |  | 8 (2.7%) | 119 (40.9%) | 77 (26.5%) | 5 (1.7%) | 82 (28.2%) |  | 8 (2.7%) | 59 (20.3%) | 9 (3.1%) | 215 (73.9%) |  | 143 (49.1%) | 125 (43.0%) | 23 (7.9%) |
|  | Overall | (N=26273) |  | 614 (2.3%) | 9831 (37.4%) | 6753 (25.7%) | 337 (1.3%) | 8738 (33.3%) |  | 879 (3.3%) | 5059 (19.3%) | 407 (1.5%) | 19928 (75.8%) |  | 13102 (49.9%) | 10177 (38.7%) | 2994 (11.4%) |
| **Mitogen-activated protein kinase (MEK) inhibitors** | | | | | | | | | | | | | | | | | |
|  | NO_target_PT | (N=7825) |  | 575 (7.3%) | 2117 (27.1%) | 1573 (20.1%) | 45 (0.6%) | 3515 (44.9%) |  | 476 (6.1%) | 1944 (24.8%) | 279 (3.6%) | 5126 (65.5%) |  | 2650 (33.9%) | 2709 (34.6%) | 2466 (31.5%) |
|  | target_PT | (N=87) |  | 7 (8.0%) | 33 (37.9%) | 20 (23.0%) | 0 (0%) | 27 (31.0%) |  | 7 (8.0%) | 37 (42.5%) | 2 (2.3%) | 41 (47.1%) |  | 32 (36.8%) | 40 (46.0%) | 15 (17.2%) |
|  | Overall | (N=7912) |  | 582 (7.4%) | 2150 (27.2%) | 1593 (20.1%) | 45 (0.6%) | 3542 (44.8%) |  | 483 (6.1%) | 1981 (25.0%) | 281 (3.6%) | 5167 (65.3%) |  | 2682 (33.9%) | 2749 (34.7%) | 2481 (31.4%) |
| **Human epidermal growth factor receptor 2 (HER2) tyrosine kinase inhibitors** | | | | | | | | | | | | | | | | | |
|  | NO_target_PT | (N=10272) |  | 4 (0.0%) | 3484 (33.9%) | 1331 (13.0%) | 42 (0.4%) | 5411 (52.7%) |  | 244 (2.4%) | 2149 (20.9%) | 123 (1.2%) | 7756 (75.5%) |  | 7466 (72.7%) | 535 (5.2%) | 2271 (22.1%) |
|  | target_PT | (N=62) |  | 0 (0%) | 24 (38.7%) | 7 (11.3%) | 1 (1.6%) | 30 (48.4%) |  | 1 (1.6%) | 12 (19.4%) | 1 (1.6%) | 48 (77.4%) |  | 51 (82.3%) | 4 (6.5%) | 7 (11.3%) |
|  | Overall | (N=10334) |  | 4 (0.0%) | 3508 (33.9%) | 1338 (12.9%) | 43 (0.4%) | 5441 (52.7%) |  | 245 (2.4%) | 2161 (20.9%) | 124 (1.2%) | 7804 (75.5%) |  | 7517 (72.7%) | 539 (5.2%) | 2278 (22.0%) |
| **Janus-associated kinase (JAK) inhibitors** | | | | | | | | | | | | | | | | | |
|  | NO_target_PT | (N=59464) |  | 104 (0.2%) | 2109 (3.5%) | 4672 (7.9%) | 521 (0.9%) | 52058 (87.5%) |  | 197 (0.3%) | 2206 (3.7%) | 213 (0.4%) | 56848 (95.6%) |  | 5439 (9.1%) | 5784 (9.7%) | 48241 (81.1%) |
|  | target_PT | (N=601) |  | 0 (0%) | 21 (3.5%) | 65 (10.8%) | 8 (1.3%) | 507 (84.4%) |  | 2 (0.3%) | 39 (6.5%) | 1 (0.2%) | 559 (93.0%) |  | 60 (10.0%) | 65 (10.8%) | 476 (79.2%) |
|  | Overall | (N=60065) |  | 104 (0.2%) | 2130 (3.5%) | 4737 (7.9%) | 529 (0.9%) | 52565 (87.5%) |  | 199 (0.3%) | 2245 (3.7%) | 214 (0.4%) | 57407 (95.6%) |  | 5499 (9.2%) | 5849 (9.7%) | 48717 (81.1%) |
| **Vascular endothelial growth factor receptor (VEGFR) tyrosine kinase inhibitors** | | | | | | | | | | | | | | | | | |
|  | NO_target_PT | (N=14367) |  | 39 (0.3%) | 5297 (36.9%) | 5870 (40.9%) | 183 (1.3%) | 2978 (20.7%) |  | 223 (1.6%) | 2587 (18.0%) | 475 (3.3%) | 11082 (77.1%) |  | 4071 (28.3%) | 9222 (64.2%) | 1074 (7.5%) |
|  | target_PT | (N=200) |  | 0 (0%) | 53 (26.5%) | 112 (56.0%) | 1 (0.5%) | 34 (17.0%) |  | 6 (3.0%) | 48 (24.0%) | 11 (5.5%) | 135 (67.5%) |  | 74 (37.0%) | 112 (56.0%) | 14 (7.0%) |
|  | Overall | (N=14567) |  | 39 (0.3%) | 5350 (36.7%) | 5982 (41.1%) | 184 (1.3%) | 3012 (20.7%) |  | 229 (1.6%) | 2635 (18.1%) | 486 (3.3%) | 11217 (77.0%) |  | 4145 (28.5%) | 9334 (64.1%) | 1088 (7.5%) |
| **Phosphatidylinositol-3-kinase (Pi3K) inhibitors** | | | | | | | | | | | | | | | | | |
|  | NO_target_PT | (N=13845) |  | 113 (0.8%) | 2642 (19.1%) | 4370 (31.6%) | 330 (2.4%) | 6390 (46.2%) |  | 197 (1.4%) | 1915 (13.8%) | 172 (1.2%) | 11561 (83.5%) |  | 8511 (61.5%) | 3905 (28.2%) | 1429 (10.3%) |
|  | target_PT | (N=79) |  | 3 (3.8%) | 11 (13.9%) | 32 (40.5%) | 5 (6.3%) | 28 (35.4%) |  | 3 (3.8%) | 10 (12.7%) | 1 (1.3%) | 65 (82.3%) |  | 39 (49.4%) | 32 (40.5%) | 8 (10.1%) |
|  | Overall | (N=13924) |  | 116 (0.8%) | 2653 (19.1%) | 4402 (31.6%) | 335 (2.4%) | 6418 (46.1%) |  | 200 (1.4%) | 1925 (13.8%) | 173 (1.2%) | 11626 (83.5%) |  | 8550 (61.4%) | 3937 (28.3%) | 1437 (10.3%) |
| **Other protein kinase inhibitors** | | | | | | | | | | | | | | | | | |
|  | NO_target_PT | (N=163124) |  | 1928 (1.2%) | 44358 (27.2%) | 54591 (33.5%) | 2129 (1.3%) | 60118 (36.9%) |  | 3398 (2.1%) | 28097 (17.2%) | 3004 (1.8%) | 128625 (78.9%) |  | 59488 (36.5%) | 90030 (55.2%) | 13606 (8.3%) |
|  | target_PT | (N=2368) |  | 15 (0.6%) | 582 (24.6%) | 1114 (47.0%) | 53 (2.2%) | 604 (25.5%) |  | 83 (3.5%) | 575 (24.3%) | 76 (3.2%) | 1634 (69.0%) |  | 935 (39.5%) | 1272 (53.7%) | 161 (6.8%) |
|  | Overall | (N=165492) |  | 1943 (1.2%) | 44940 (27.2%) | 55705 (33.7%) | 2182 (1.3%) | 60722 (36.7%) |  | 3481 (2.1%) | 28672 (17.3%) | 3080 (1.9%) | 130259 (78.7%) |  | 60423 (36.5%) | 91302 (55.2%) | 13767 (8.3%) |
