# Supplementary material for: Central nervous system hemorrhage related to protein kinase inhibitors: A real-world pharmacovigilance case-control study and genetic correlation study
Source: Medicine (Baltimore). 2026 Nov 6;105(45):e51382. doi: 10.1097/MD.0000000000051382 (PMC13747402; doi:10.1097/MD.0000000000051382)
Supplement: Supplementary file 7 [file medi-105-e51382-s007.docx]

**Supplementary file7. The details on MR analyses**

| **Exposures on outcomes** | **Methods** | **nSNPs** | **Beta** | **SE** | **P-value** | **Q** | **Q-value** |
| --- | --- | --- | --- | --- | --- | --- | --- |
| Chronic myeloid leukemia on ICH | Inverse variance weighted | 32 | 0.025 | 0.028 | 0.362 | 22.836 | 0.855 |
|  | MR Egger | 32 | 0.091 | 0.070 | 0.190 | 21.712 | 0.864 |
|  | Weighted median | 32 | 0.022 | 0.040 | 0.575 |  |  |
|  | Simple mode | 32 | 0.032 | 0.071 | 0.661 |  |  |
|  | Weighted mode | 32 | 0.017 | 0.066 | 0.800 |  |  |
| Lymphoid leukaemia on ICH | Inverse variance weighted | 69 | -0.002 | 0.044 | 0.969 | 74.989 | 0.262 |
|  | MR Egger | 69 | 0.114 | 0.094 | 0.233 | 72.923 | 0.290 |
|  | Weighted median | 69 | -0.028 | 0.062 | 0.651 |  |  |
|  | Simple mode | 69 | -0.073 | 0.155 | 0.640 |  |  |
|  | Weighted mode | 69 | -0.078 | 0.157 | 0.619 |  |  |
| Gastrointestinal stromal tumor on ICH | Inverse variance weighted | 33 | 0.003 | 0.028 | 0.927 | 26.057 | 0.761 |
|  | MR Egger | 33 | 0.005 | 0.066 | 0.939 | 26.055 | 0.719 |
|  | Weighted median | 33 | 0.006 | 0.040 | 0.890 |  |  |
|  | Simple mode | 33 | 0.037 | 0.072 | 0.611 |  |  |
|  | Weighted mode | 33 | 0.021 | 0.066 | 0.751 |  |  |
| Mantle cell lymphoma on ICH | Inverse variance weighted | 24 | -0.017 | 0.031 | 0.586 | 23.537 | 0.430 |
|  | MR Egger | 24 | -0.013 | 0.057 | 0.825 | 23.530 | 0.372 |
|  | Weighted median | 24 | -0.016 | 0.044 | 0.723 |  |  |
|  | Simple mode | 24 | -0.084 | 0.081 | 0.313 |  |  |
|  | Weighted mode | 24 | -0.028 | 0.079 | 0.721 |  |  |
| Chronic myeloid leukemia on subdural hemorrhage | Inverse variance weighted | 34 | -0.029 | 0.041 | 0.479 | 19.020 | 0.975 |
|  | MR Egger | 34 | -0.100 | 0.086 | 0.253 | 18.136 | 0.977 |
|  | Weighted median | 34 | -0.062 | 0.060 | 0.298 |  |  |
|  | Simple mode | 34 | -0.098 | 0.120 | 0.420 |  |  |
|  | Weighted mode | 34 | -0.092 | 0.088 | 0.307 |  |  |
| Lymphoid leukaemia on subdural hemorrhage | Inverse variance weighted | 56 | -0.110 | 0.075 | 0.141 | 48.610 | 0.716 |
|  | MR Egger | 56 | -0.028 | 0.139 | 0.843 | 48.119 | 0.700 |
|  | Weighted median | 56 | -0.197 | 0.105 | 0.061 |  |  |
|  | Simple mode | 56 | -0.331 | 0.249 | 0.191 |  |  |
|  | Weighted mode | 56 | -0.316 | 0.212 | 0.142 |  |  |
| Gastrointestinal stromal tumor on subdural hemorrhage | Inverse variance weighted | 32 | -0.009 | 0.046 | 0.846 | 33.934 | 0.328 |
|  | MR Egger | 32 | -0.047 | 0.110 | 0.674 | 33.773 | 0.290 |
|  | Weighted median | 32 | 0.023 | 0.063 | 0.718 |  |  |
|  | Simple mode | 32 | 0.044 | 0.120 | 0.713 |  |  |
|  | Weighted mode | 32 | 0.039 | 0.106 | 0.713 |  |  |
| Mantle cell lymphoma on subdural hemorrhage | Inverse variance weighted | 23 | -0.056 | 0.046 | 0.224 | 17.797 | 0.718 |
|  | MR Egger | 23 | -0.025 | 0.084 | 0.767 | 17.597 | 0.674 |
|  | Weighted median | 23 | -0.018 | 0.067 | 0.791 |  |  |
|  | Simple mode | 23 | 0.004 | 0.113 | 0.975 |  |  |
|  | Weighted mode | 23 | -0.005 | 0.097 | 0.962 |  |  |
